# Paracentral Acute Middle Maculopathy Associated With Dengue Fever

**DOI:** 10.7759/cureus.100159

**Published:** 2025-12-26

**Authors:** Salil Mehta

**Affiliations:** 1 Ophthalmology, Lilavati Hospital, Mumbai, IND

**Keywords:** dengue fever, maculopathy, optical coherence tomography, paracentral acute middle maculopathy, retinal disease

## Abstract

Newer imaging technique systems have led to an increased interest in the anatomical localization of retinal disease. The clinical and optical coherence tomography (OCT) features of paracentral acute middle maculopathy (PAMM) have been increasingly described and are classically defined by a hyperreflective band at the level of the inner nuclear layer (INL). Symptoms typically include blurred central vision with a paracentral scotoma. Potential causes include retinal vascular diseases and systemic disorders.

We present the clinical and OCT findings of a 33-year-old male patient with PAMM following dengue fever. The patient presented with a five-day history of fever, chills, severe body aches, and a generalized body rash. On day 1, he had undergone a series of investigations to determine the cause, and the test results were positive for dengue fever (Rapid NS antigen 1 45.47 S/Co (positive > 1.0 S/Co). On day five, he noticed bleeding from his gums, which correlated to a drop in his platelet count to 77,000/mm^3^. At this time, he complained of a scotoma in front of the right eye with mildly blurred vision. His visual acuity was 6/12, N8 in the right eye and 6/6, N6 in the left eye. Slit lamp examination of the anterior segment was normal in either eye. Dilated fundus evaluation revealed multiple grayish-white areas of retinitis in the superior macula with associated hemorrhages. An OCT scan revealed multiple areas of hyperreflectivity in the inner retina, primarily from the inner nuclear and inner plexiform layers. He was treated with oral prednisolone 40 mg/day with tapering every week. At day seven, there was a significant reduction in his symptoms with an increase in the visual acuity to 6/9, N6, and a decrease in the size of the retinal lesions and inner retina hyper reflectivity

A wide spectrum of ocular complications has been described in dengue. Common anterior segment lesions include conjunctival petechiae, subconjunctival hemorrhage, or anterior uveitis. Common posterior segment findings include retinal hemorrhages, posterior uveitis, and several presentations of dengue maculopathy. PAMM following dengue fever has only rarely been reported. We hypothesize that dengue-induced local inflammatory or hemoconcentration processes may have led to deep capillary plexus (DCP) circulatory compromise, leading to ischemia, especially in the area supplied by the DCP. The onset of a central scotoma may suggest the onset of PAMM, and this requires a comprehensive ocular evaluation, and if required, an OCT test to rule out this possibility. Ophthalmologists should be aware of this differential diagnosis.

## Introduction

A number of lesions, termed white dot syndromes, marked by multiple, white-to-yellow inflammatory lesions, located at the level of the outer retinal layers and the choroid, have been reported in the literature. These include acute posterior multifocal placoid pigment epitheliopathy, multiple evanescent white dot syndrome, multifocal choroiditis with panuveitis, and acute zonal occult outer retinopathy [1]. One phenotype, acute macular neuroretinopathy (AMN), first described by Bos and Deutman, is classically described as reddish-brown, wedge-shaped lesions in the macular area. In contrast to other white dot syndromes, which are primarily inflammatory, AMN is thought to be a consequence of the noninflammatory ischemia of the retinal capillaries, usually the outer plexiform layer (OPL) and outer nuclear layer (ONL) [2].

Paracentral acute middle maculopathy (PAMM) is now increasingly being recognized as another ischemic pathology characterised by the appearance of hyperreflective lesions at the level of the inner nuclear layer (INL), more superficial than the lesions seen in AMN. Symptoms typically include blurred central vision with a paracentral scotoma. Potential causes include retinal vascular diseases and systemic disorders, including diabetic retinopathy and arterial and venous occlusions [2]. PAMM following dengue fever has only rarely been reported. We present the clinical and optical coherence tomography (OCT) findings of a 33-year-old male patient with PAMM following dengue fever.

## Case presentation

A 33-year-old male presented with a five-day history of fever, chills, severe body ache, and a generalized body rash. On day one, he had undergone a series of investigations for the cause, and test results were positive for dengue fever (Rapid NS antigen 1 45.47 S/Co (positive > 1.0 S/Co) with dengue immunoglobulin (Ig)M positivity. At this time, his platelet count was 123,000 (normal 200,000 to 500,000/mm3). Test results for chikungunya infection were negative.

On day five, he noticed bleeding from his gums, which correlated to a drop in his platelet count to 77,000/mm^3^. He was admitted for observation. On admission, he was afebrile with normal examination findings of the cardiovascular, respiratory, and nervous systems. The salient hematological test results on day one/two and day five are shown in Table 1. 

**Table 1:** Salient hematological parameters on day one/two and day five

Parameter	Values	Normal Range
Day1 - Day 2		
Hemoglobin	16.0 gm/dl	13.0-17.0 gm/dl
Hematocrit	48.2%	40-50%
White Cell Count	2530/mm^3^	4000-10,000 /mm^3^
Platelet Count	122,000/mm^3^	150,000-410,000 /mm^3^
Day 5		
Hemoglobin	14.50 gm/dl	13.0-17.0 gm/dl
Hematocrit	43.30%	
White Cell Count	5700	4000-10,000/mm^3^
Absolute Neutrophil Count	1100	2000-7000
Absolute Lymphocyte Count	3800	1000-3000
Absolute Eosinophil Count	0.0	20-500
Absolute Basophil Count	0.0	20-100
Neutrophils	20.00	40.0-80.0%
Lymphocytes	66.00	20.0-40.0%
Monocytes	14.00	2.0-10.0%
Eosinophils	0.0	1.0-6.0%
Basophils	0.0	1.0-2.0%
Platelet Count	77,000	150,000-410,000/mm^3^

**Figure 1:**
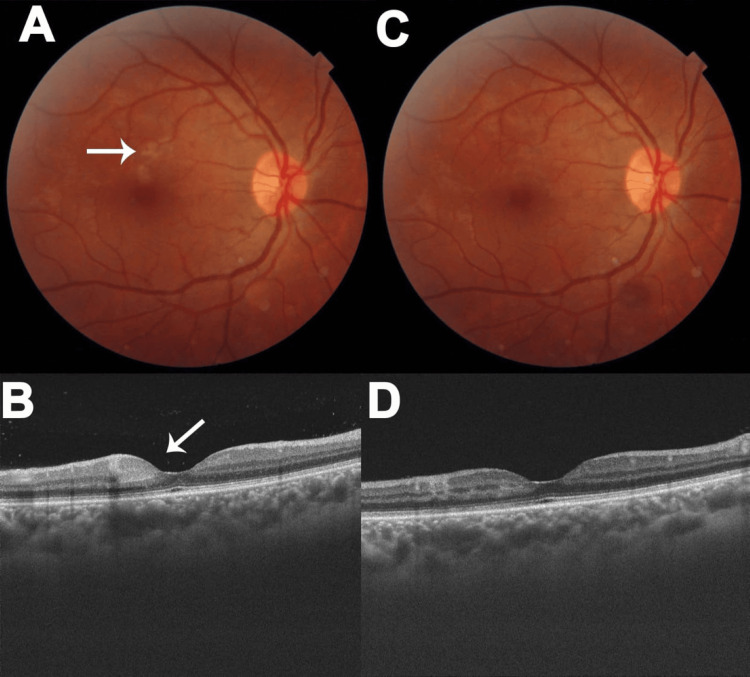
Optical coherence tomography of dilated right eye fundus A) Fundus photograph of the right eye, showing grayish-white retinal lesions (arrow); B) OCT scan showing hyperreflectivity of the inner nuclear and inner plexiform layers; C) Fundus photograph on day five showing reduction of the retinal lesions; D) OCT scan showing reduction of the  hyperreflectivity

At this time, he complained of a scotoma in front of the right eye with mildly blurred vision. His visual acuity was 6/12, N8 in the right eye and 6/6, N6 in the left eye. Slit lamp examination of the anterior segment was normal in either eye. The clinical and OCT findings of a dilated fundus examination are shown in Figure 1. This revealed multiple grayish-white areas of retinitis in the superior macula of the right eye, with associated hemorrhages (Figure 1A). An OCT scan revealed multiple areas of hyperreflectivity in the inner retina, primarily from the inner nuclear and inner plexiform layers (Figure 1B). Assuming an immune-mediated etiology, he was treated empirically with oral prednisolone 40 mg/day with tapering every week. At day seven, there was a significant reduction in his symptoms, with an increase in visual acuity to 6/9, N6, and a decrease in the size of the retinal lesions (Figure 1C) and the inner retina hyperreflectivity (Figure 1D). The left eye was normal on both occasions. 

## Discussion

PAMM was initially described as a variant of AMN. Rahimy et al. were the first to describe PAMM and its clinical and OCT findings, in the form of hyper-reflective lesions in the middle retina, usually located in the INL or outer plexiform layer junction (OPL) [3]. Several subsequent studies have expanded our understanding of PAMM. In a large retrospective study, Limoli et al. identified 78 patients from a diverse background. Simultaneous retinal vascular disease occurred in 38 patients (16 with arterial occlusion and 20 with venous occlusion) as compared to 40 patients without. The average age at presentation was 54.5 years, with a largely male cohort. PAMM was seen as an isolated phenomenon or secondary to retinal vascular disease. In the absence of retinal vascular disease, patients tended to have an increased prevalence of systemic vasculopathies, suggesting the pivotal role of ischemia [4]. 

The capillary plexuses of the retina are organized into three layers: the superficial capillary plexus (SCP), intermediate capillary plexus (ICP), and deep capillary plexus (DCP). These plexuses are independently autoregulated, thus ensuring an adequate blood flow to all parts of the inner and middle retina. The DCP perfuses the middle retina, but its perfusion pressure is lower compared to the SCP and the ICP. Additionally, the high metabolic demand of the outer nuclear layer has led to the development of a watershed zone concept that renders the DCP uniquely vulnerable to hemodynamic dysfunction, with a subsequent ischemia of the middle retina, specifically, the inner nuclear layer, with a possible extension to the inner plexiform layer. The inner nuclear layer thinning following PAMM tends to confirm this hypothesis [5].

Dengue fever is a viral fever that is caused by one of the four serotypes of the dengue virus, which are members of the flaviviridae family. An estimated 100-400 million infections occur every year, with the disease being endemic in parts of Africa, the Americas, and Southeast Asia. Numerous hematological abnormalities occur in acute dengue infection, including thrombocytopenia, leukopenia, and increased hematocrit. Of these, a rise in hematocrit is an indicator of plasma leakage into the extravascular spaces and the subsequent reduction of intravascular volume, which could lead to circulatory disturbances. This could be widespread ischemia or localized organ ischemia [6].

A wide spectrum of ocular complications has been described in dengue. The pathogenesis of dengue fever-related ocular manifestations is now thought to be immune-mediated. Most lesions appear approximately a week after the appearance of fever. In one study, as many as 24.1% of inpatients noted visual symptoms, commonly blurred vision or scotomas [7]. Potential mechanisms include immune complex deposition, complement activation, and autoantibody formation. Interestingly, the dengue virus may incite autoimmune reactions against numerous ocular tissues, such as the retina, retinal pigment epithelium, or the choroid. Commonly seen lesions include subconjunctival hemorrhage, anterior uveitis, intermediate uveitis, and posterior uveitis (retinitis, chorioretinitis, and neuroretinitis) or retinal vasculitis [7].

Retinal vasculitis is commonly seen and usually co-exists with retinitis or retinochoroiditis. The use of Fundus fluorescein angiography (FFA), led to the discovery of the classical findings including early vascular blockage or delayed perfusion, followed by mid- and late-phase vessel wall hyperfluorescence and localized areas of blocked fluorescence. The increasing use of OCT permitted the characterization of AMN with the identification of disruptions of the ellipsoid zone, external limiting membrane, and interdigitation zones and hyperreflectivity of the ONL and the OPL [8]. 

PAMM following dengue fever has only rarely been reported. In a single case report, Ferreira et al. describe a 19-year-old patient with symptoms suggestive of dengue fever. Dilated fundus evaluation revealed bilateral optic disc edema and deep hemorrhages. An OCT evaluation revealed a hyperreflectivity involving the outer nuclear and plexiform layers. Following a course of oral steroids, the visual acuity significantly improved with residual INL thinning [9].

The patient we describe was diagnosed with dengue fever based on a positive rapid NS1 and positive IgM titers. Thrombocytopenia was noticed on admission, but this rapidly improved on supportive therapy. His hematocrit was normal on day 1 and day 5, suggesting the absence of a profound systemic plasma leakage. The retinal lesions were in the middle retina layers of the peripheral macula rather than in the central macula. The clinical phenotype would be consistent with a diagnosis of PAMM.

We hypothesize that dengue-induced local inflammatory or hemoconcentration processes may have led to DCP circulatory compromise, leading to ischemia, especially in the area supplied by the DCP. Chanthick and co-workers have suggested that a viral endotheliitis may lead to the production of proinflammatory cytokines and chemokines and consequent leakage. Transcellular hyperpermeability is also hypothesized to be important in the pathogenesis of plasma leakage in patients with dengue fever [10]. The findings we describe are those detected during his hospitalization. A further follow-up would have led to a changed OCT picture, which would shed further light on the long-term evolution of these lesions. This remains an area for future study. 

## Conclusions

Dengue fever is a common infectious disease with multiple hematological and ocular manifestations. While many manifestations, especially the hemorrhagic lesions, are well described, PAMM has only recently been described. While an ophthalmic evaluation is not yet part of the standard protocol for these patients, we suggest adopting it, as asymptomatic patients or those with subtle symptoms may be overlooked. In particular, the onset of a central scotoma may suggest the onset of PAMM, and this is an OCT test to rule out this possibility. Ophthalmologists should be aware of this entity and its differential diagnosis.
